# Environmental Effects via Frozen Density Embedding in Real-Time Time-Dependent Dirac–Kohn–Sham Theory: Solvation of Lead Halides

**DOI:** 10.1021/acs.jctc.5c01980

**Published:** 2026-02-23

**Authors:** Matteo De Santis, Edoardo Mosconi, Leonardo Pacifici, Valérie Vallet, André Severo Pereira Gomes, Loriano Storchi, Leonardo Belpassi

**Affiliations:** † 129786 Univ. Lille, CNRS, UMR 8523-PhLAM-Physique des Lasers Atomes et Molécules, F-59000 Lille, France; ‡ Istituto di Scienze e Tecnologie Chimiche (SCITEC), Consiglio Nazionale delle Ricerche c/o Dipartimento di Chimica, Biologia e Biotecnologie, Università degli Studi di Perugia, Via Elce di Sotto 8, 06123 Perugia, Italy; § Dipartimento di Farmacia, Università degli Studi ‘G. D’Annunzio’, Via dei Vestini 31, 66100 Chieti, Italy

## Abstract

Accurately describing the electronic properties of heavy-element molecular systems in complex environments is essential for advancing technologies such as optoelectronics and solar cells. However, achieving accurate predictions remains challenging because both relativistic and electron correlation effects must be considered equally, along with interactions involving other species in the complex environment (e.g., solvent). This paper extends our real-time time-dependent Dirac-Kohn–Sham (rt-TDDKS) implementation in PyBERTHA-RT to include environmental effects using the “uncoupled” Frozen-Density-Embedding (FDE) scheme, where only the active subsystem evolves dynamically in time. This adaptation utilizes existing FDE functionality within the PyEmbed module of the PyADF scripting framework. The native Python APIs of PyBERTHA-RT and PyADF provide an ideal environment for development, enhancing readability and reusability. We demonstrate that the FDE potential maintains the numerical stability of the active subsystem’s density matrix propagation. Illustrative results for lead halides (PbCl_2_ and PbI_2_) in γ-butyrolactone (GBL) solution show the effects of increasing solvent molecules on absorption spectra. This case study demonstrates the new implementation’s applicability to realistic systems, offering a basis for studying electron dynamics in heavy-element molecules in complex environments under linear and nonlinear regimes, relevant to perovskite precursor chemistry.

## Graphical abstract

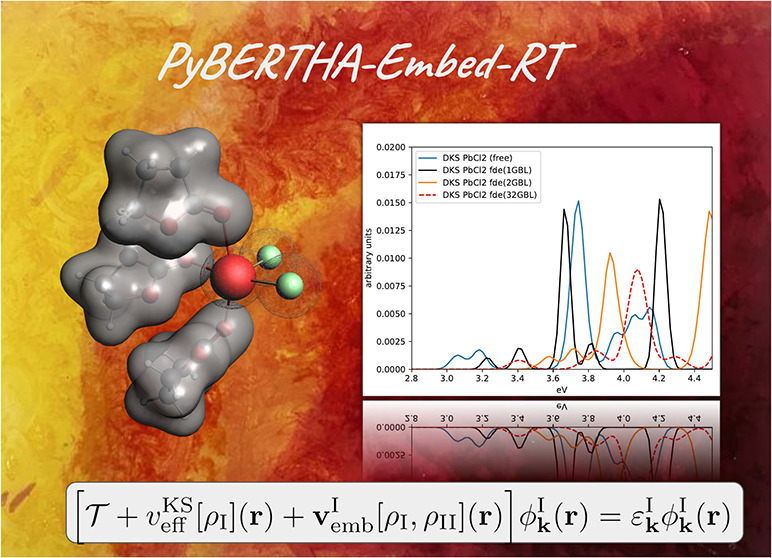

## 1 Introduction

The interaction between matter and radiation underlies a wide range of phenomena, spanning weak-field processes such as photoexcitation, absorption, scattering, light harvesting in dye-sensitized solar cells,^1,2^ and photoionization, as well as strong-field effects including high harmonic generation,^3,4^ optical rectification,^5,6^ multiphoton ionization,^7^ and above-threshold ionization.^8^ The advent of Free Electron Lasers (FELs) and attosecond techniques^9,10^ has opened new avenues for real-time studies of electron dynamics and chemical reactions, enabling the observation and potential control of electron motion within molecules. These approaches provide direct insights into bond breaking,^11–13^ bond formation,^14^ and ionization processes^15,16^ on ultrafast time scales.

Real-time time-dependent electronic structure theory, which directly solves the time-dependent equations of electronic motion, is particularly well suited for studying molecular responses and electronic dynamics. This approach is capable of capturing complex nonlinear phenomena and the effects of shaped external fields, making it essential for applications in quantum optimal control theory.^17^ Significant progress in these techniques has been reviewed by Goings et al.,^18^ with real-time time-dependent density functional theory (rt-TDDFT) emerging as a method of choice due to its favorable balance between accuracy and computational efficiency.^19–30^

Early rt-TDDFT simulations primarily focused on isolated systems, but electronic and optical properties are often strongly influenced by environmental polarization, making isolated-molecule models insufficient. To address this limitation, rt-TDDFT has been combined with QM/MM^30,31^ and polarizable continuum models (PCM)^32–34^ to include environmental effects at reduced computational cost. These approaches have subsequently been extended to account for nonequilibrium solvent responses^35–38^ and nonequilibrium cavity-field polarization in homogeneous dielectrics.^39^ To move beyond classical descriptions of the environment, Koh et al.^40^ integrated rt-TDDFT with block-orthogonalized (BO) Manby-Miller theory,^41^ enabling faster simulations and the explicit inclusion of solvation effects. This framework was later extended by some of the present authors to study environmental influences on X-ray absorption spectra of halogen anions in water.^42^ A quantum mechanical alternative for incorporating environmental effects is provided by the frozen-density embedding (FDE) scheme, a DFT-in-DFT framework that partitions a larger Kohn–Sham system into smaller, coupled subsystems.^43–45^ By treating all subsystems quantum mechanically, FDE not only offers computational advantages but also provides detailed insight into subsystem properties and intersystem couplings.^46^ Neugebauer^47,48^ introduced coupled FDE, a subsystem TDDFT formulation based on linear response that eliminated some of the approximations present in earlier TDDFT-FDE implementations and allowed great flexibility in controlling which subsystems are allowed to respond to the time-dependent perturbations. More recently, this approach has been further extended^49,50^ to address charge-transfer excitations, leveraging an exact FDE framework.^51–55^ Krishtal et al.^46^ developed a real-time DFT subsystem formulation, extending FDE to rt-TDDFT for the first time by updating the embedding potential and evolving Kohn–Sham subsystems simultaneously. This work showed that, at least within an implementation based on plane waves and periodic boundary conditions using pseudopotentials, the method is numerically stable. An “uncoupled” scheme can approximate the full response of the system by restricting the density response to a single active subsystem while freezing all others. Even in this uncoupled formulation, the embedding potential remains time-dependent in real-time TDDFT-FDE and must therefore be recalculated during the propagation. This approach restricts time evolution to the active subsystem while still effectively incorporating environmental effects. Uncoupled FDE has been shown to reproduce supermolecular results in linear-response TDDFT, even in the presence of hydrogen bonding, provided that excitation couplings between subsystems are negligible.^28,46^ More recently, De Santis et al.^56^ extended the nonrelativistic real-time time-dependent Kohn–Sham formulation based on localized Gaussian basis set functions and implemented it within the Psi4NumPy framework,^57,58^ including real-time embedded TDDFT schemes that combine the FDE and BOMME approaches.^59^ Additional recent implementations can be found in refs 60,61 .

For molecular systems containing heavy elements, the accurate inclusion of relativistic effects is mandatory. The rt-TDDFT scheme has been extended to incorporate high-level relativistic corrections. In particular Repisky et al.^62^ pioneered relativistic real-time TDDFT for atomic and molecular systems using the four-component Dirac-Kohn–Sham (DKS) method (rt-TDDKS), while nearly concurrently Goings et al.^63^ developed X2C Hamiltonian-based electron dynamics for UV/vis spectral calculations. More recently, a rt-TDDKS implementation (PyBERTHA-RT) leveraging modern software engineering practices, including interlanguage communication between Python, C, and Fortran was presented by some of us.^64,65^ This implementation is based on the recently developed PyBERTHA,^64–67^ which serves as the Python API for the relativistic four-component BERTHA code,^68–70^ and has been subsequently been extended to exploit GPU-based acceleration technology.^71^

Recently, Olejniczak et al.^72^ reviewed strategies to efficiently include environmental effects into accurate relativistic Hamiltonians via embedding theories. While approaches such as the Polarizable Continuum Model (PCM) can account for such effects within real-time Dirac–Kohn–Sham (rt-TDDKS) simulations, the use of quantum embedding approaches such as FDE in combination with rt-TDDKS has not yet been reported. A noteworthy recent development is an implementation in Turbomole, based on a two-component (2c) formalism for the inclusion of spin–orbit coupling, which has also been extended to periodic systems.^61^

In this work, we extend the rt-TDDKS method formulated with relativistic G-spinor localized basis functions, to the FDE framework. We take advantage of our recent implementation for ground-state DKS calculations (PyBERTHA-Embed),^73^ specifically in its uncoupled variant (uFDE). A unified Python-based framework has been developed to enable efficient interoperability between the rt-TDDKS implementation in PyBERTHA^64,65^ and the PyADF API^74^ and PyEmbed module,^56,73^ which are used to define the basic procedures for the embedding scheme. A detailed description of the PyEmbed module has been recently presented by Focke et al.^75^ For clarity, we refer to the original rt-TDDKS implementation as *PyBERTHA-RT*, and to its extension to the uFDE subsystem framework presented here as *PyBERTHA-Embed-RT*.

To demonstrate the capability of the new implementation to tackle large-scale systems, we apply it to investigate the influence of a solvent environment composed of γ-butyrolactone (GBL) molecules on the valence spectra of the perovskite precursors PbCl_2_ and PbI_2_. These systems were previously studied by some of us^76^ using frequency-domain TDDFT calculations based on structures obtained from ab initio molecular dynamics (AIMD) simulations. Here, the focus is on assessing the numerical stability of the implementation and its accuracy with respect to both auxiliary fitting and G-spinor basis sets; consequently only structures from a single AIMD snapshot are considered. Although a single structure is insufficient to yield spectra directly comparable to experiment, it allows us to distinguish solvent effects arising from the nearest neighbors to those associated with the second solvation shell and beyond. To this end, we consider models containing 1, 2, and 3 GBL molecules (the latter corresponding to roughly the average GBL coordination number for the first solvation shell^76^ for PbCl_2_ and PbI_2_) as well as the full system containing all 32 GBL molecules in the simulation supercell.^76^

The manuscript is organized as follows. In Section 2, we review the FDE formalism, its extension to rt-TDDKS, and the use of density fitting, with emphasis on code interoperability among PyBERTHA,^64^ XCFun,^77^ PyADF,^74^ and the PyEmbed module.^56,73,75^ Section 3 presents the results of solvation effects in the absorption spectra of PbCl_2_ and PbI_2_, and Section 4 provides conclusions and discusses perspectives.

## 2 Theory and Implementation

In this section, we briefly review the theoretical foundations of the FDE scheme, the basic equations of the Dirac-Kohn–Sham (DKS) method, and its extension to the real-time time-dependent DKS (rt-TDDKS) method as implemented in the BERTHA code using its Python API (PyBERTHA).^64,65^ We also describe the new rt-TDDKS-uFDE method, focusing on the use of the density fitting scheme, previously applied by some of us to the electronic ground state using the DKS-uFDE scheme,^73^ which accelerates the calculation of the FDE potential matrix representation for the active subsystem. Finally, we illustrate the general implementation strategy, which is greatly facilitated by BERTHA’s Python API. This framework provides flexible code development, taking advantage of Python’s reusability and portability, and enables interoperability with other FDE implementations available through the PyADF and PyEmbed modules.

### 2.1 Subsystem DFT and Frozen Density Embedding Formulation

The original concept of subsystem DFT can be traced to the work of Senatore and Subbaswamy,^78^ with a formal derivation provided by Cortona.^79^ Within the linear-response framework, Casida and Wesolowski proposed a TDDFT extension^80^ of the FDE scheme. In 2014, Jacob and Neugebauer presented a detailed theoretical analysis,^44^ and very recently published an update, including new areas of application.^81^ We refer interested readers to these comprehensive reviews.

The whole system is divided into *N* subsystems, and the total density ρ_tot_(***r***) is expressed as the sum of the electron densities of the various subsystems [i.e., ρ_*a*_(***r***) (*a* = 1,..., *N*)]. In the simplest case of a single subsystem of interest (I) interacting with an environment (II), one can consider the total density as partitioned into two contributions as

1 $$\rho_{\mathrm{tot}}\left(r\right)=\rho_{I}\left(r\right)+\rho_{\mathrm{II}}\left(r\right)$$

The total energy of the system can then be written as

2 $$E_{\mathrm{tot}}\left[\rho_{I},\rho_{\mathrm{II}}\right]=E_{I}\left[\rho_{I}\right]+E_{\mathrm{II}}\left[\rho_{\mathrm{II}}\right]+E_{\mathrm{int}}\left[\rho_{I},\rho_{\mathrm{II}}\right]$$

with the energy of each subsystem (*E*_*i*_[ρ_*i*_], with *i* = I, II) given according to the usual definition in DFT as

$$E_{i}\left[\rho_{i}\right]=\int \rho_{i}\left(r\right)v_{\mathrm{nuc}}^{i}\left(r\right)d^{3}r+\frac{1}{2}\iint \frac{\rho_{i}\left(r\right)\rho_{i}\left(r^{\prime }\right)}{\left|r-r^{\prime }\right|}d^{3}rd^{3}r^{\prime }+E_{\mathrm{xc}}\left[\rho_{i}\right]+T_{s}\left[\rho_{i}\right]+E_{\mathrm{nuc}}^{i}$$ 3

In the above expression, *v*_nuc_^*i*^(***r***) is the nuclear potential due to the set of atoms which defines the *i*-th subsystem and *E*_nuc_^*i*^ is the related nuclear repulsion energy. *T*_*s*_[ρ_*i*_]­is the kinetic energy of the auxiliary noninteracting system, which is, within the Kohn–Sham (KS) approach, commonly evaluated using the KS orbitals. The interaction energy is given by the expression

4 $$E_{\mathrm{int}}\left[\rho_{I},\rho_{\mathrm{II}}\right]=\int \rho_{I}\left(r\right)v_{\mathrm{nuc}}^{\mathrm{II}}\left(r\right)d^{3}r+\int \rho_{\mathrm{II}}\left(r\right)v_{\mathrm{nuc}}^{I}\left(r\right)d^{3}r+E_{\mathrm{nuc}}^{I,\mathrm{II}}+\iint \frac{\rho_{I}\left(r\right)\rho_{\mathrm{II}}\left(r^{\prime }\right)}{\left|r-r^{\prime }\right|}d^{3}rd^{3}r^{\prime }+E_{\mathrm{xc}}^{\mathrm{nadd}}\left[\rho_{I},\rho_{\mathrm{II}}\right]+T_{s}^{\mathrm{nadd}}\left[\rho_{I},\rho_{\mathrm{II}}\right]$$

with *v*_nuc_^I^ and *v*_nuc_^II^ the nuclear potentials due to the set of atoms associated with the subsystem I and II, respectively. The repulsion energy for nuclei belonging to different subsystems is described by the *E*_nuc_^I,II^ term. The nonadditive contributions are defined as

5 $$X^{\mathrm{nadd}}\left[\rho_{I},\rho_{\mathrm{II}}\right]=X\left[\rho_{I}+\rho_{\mathrm{II}}\right]-X\left[\rho_{I}\right]-X\left[\rho_{\mathrm{II}}\right]$$

with X = *E*_xc_, *T*_*s*_. These terms arise because both exchange-correlation and kinetic energy, in contrast to the Coulomb interaction, are not linear functionals of the density.

The electron density of a given fragment (ρ_I_ or ρ_II_ in this case) can be determined by minimizing the total energy functional (eq 2) with respect to the density of the fragment, while keeping the density of the other subsystem frozen. This procedure is the essence of the FDE scheme (first introduced by Wesolowski and Warshel in ref 82.) and leads to a set of Kohn–Sham-like equations, one for each subsystem.

6 $$\left[T+v_{\mathrm{eff}}^{\mathrm{KS}}\left[\rho_{I}\right]\left(r\right)+v_{\mathrm{emb}}^{I}\left[\rho_{I},\rho_{\mathrm{II}}\right]\left(r\right)\right]\phi_{k}^{I}\left(r\right)=\varepsilon_{k}^{I}\phi_{k}^{I}\left(r\right)$$

which are coupled by the embedding potential term *v*_emb_^I^(***r***), which carries all dependence on the other fragment’s density. In this equation, *v*_eff_^KS^[ρ_I_]­(***r***) is the KS potential calculated on the basis of the density of subsystem I only, whereas the embedding potential takes into account the effect of the other subsystem (which we consider here as the complete environment). Here T denotes the kinetic energy operator, which in a nonrelativistic framework has the form −∇^2^/2, whereas for a relativistic framework (Dirac-Kohn–Sham theory) is *c* **α**·**p** (see discussion below). We also note that, in the relativistic framework, the FDE expressions above correspond to the case in which an external vector potential is absent. Further details for their generalization can be found in ref 83. In the framework of FDE theory, *v*_emb_^I^(***r***) is explicitly given by

7 $$v_{\mathrm{emb}}^{I}\left[\rho_{I},\rho_{\mathrm{II}}\right]\left(r\right)=\frac{\delta E_{\mathrm{int}}\left[\rho_{I},\rho_{\mathrm{II}}\right]}{\delta \rho_{I}\left(r\right)}=v_{\mathrm{nuc}}^{\mathrm{II}}\left(r\right)+\int \frac{\rho_{\mathrm{II}}\left(r^{\prime }\right)}{\left|r-r^{\prime }\right|}d^{3}r^{\prime }+\frac{\delta E_{\mathrm{xc}}^{\mathrm{nadd}}\left[\rho_{I},\rho_{\mathrm{II}}\right]}{\delta \rho_{I}\left(r\right)}+\frac{\delta T_{s}^{\mathrm{nadd}}\left[\rho_{I},\rho_{\mathrm{II}}\right]}{\delta \rho_{I}\left(r\right)}$$

where the nonadditive exchange-correlation and kinetic energy contributions are defined as the difference between the associated exchange-correlation and kinetic potentials defined using ρ_tot_(***r***) and ρ_I_(***r***). For both potentials, one needs to account for the fact that only the density is known for the total system so that potentials that require input in the form of KS orbitals are, in practice, prohibited (as orthogonal KS orbitals are only obtained for each subsystems, not for the whole system). For the exchange-correlation potential, one may make use of accurate density functional approximations and its quality is therefore similar to that of ordinary KS. The potential for the nonadditive kinetic term ( $\frac{\delta T_{s}^{\mathrm{nadd}}\left[\rho \right]}{\delta \rho_{I}\left(r\right)}$ , in eq 7) is more problematic, as less accurate orbital-free kinetic energy density functionals (KEDFs) are available for this purpose. Examples of popular functional approximations applied in this context are the Thomas-Fermi (TF) kinetic energy functional^84^ or the GGA functional PW91k.^85^ These functionals have been shown to be accurate for weakly interacting systems, including hydrogen-bonded systems, whereas their use for subsystems with a larger covalent character is problematic (see ref 51 and references therein). The search for more accurate KEDFs is a key aspect for the applicability of the FDE scheme as a general approach, including the partitioning of systems that also involve breaking covalent bonds.^86^ For a comprehensive review of recent efforts in the development of accurate KEDFs, see ref 87.

In general, the set of coupled equations that arise in the FDE scheme for the subsystems must be solved iteratively. Typically, a “freeze-and-thaw” procedure is employed, where the electron density of the active subsystem is determined while keeping the electron densities of the other subsystems frozen. The former is then frozen in turn when the electron densities of the remaining subsystems are relaxed. This process may be repeated multiple times until all subsystem densities have converged. In this case, the FDE scheme can be viewed as an alternative formulation of the conventional KS-DFT approach for large systems, and by construction, it scales linearly with the number of subsystems.

Within the ground-state SCF or real-time propagation frameworks, the implementation of FDE is efficient, as the *v*_*emb*_^*I*^(*r*) contribution can be treated as an effective one-electron potential added to the Kohn–Sham matrix. Unlike linear-response formulations, which require the explicit evaluation of nonadditive embedding kernels,^47,80,88^ the real-time scheme captures these interactions implicitly through the time-evolution of the potential. When using localized basis functions, the matrix representation of the embedding potential (**V** ^emb^) can be evaluated using numerical integration grids similar to those used for the exchange-correlation term in the KS method. This contribution is then added to the KS matrix, and the eigenvalue problem is solved in the usual self-consistent field manner. It should be noted that, regardless of whether one or multiple subsystem densities are optimized, the matrix **V** ^emb^ must be updated during the SCF procedure because it also depends on the density of the active subsystem (see eq 7).

Thanks to its flexibility, the FDE scheme has been implemented in various forms in many computational packages,^61,89–98^ based on plane waves, Slater-type functions, or Gaussian-type functions. FDE has also been implemented to treat subsystems at the fully relativistic four-component level based on the Dirac equation within the DIRAC code,^99^ and can be used with DFT and various wave function methods for both molecular properties and energies involving ground or excited electronic states.^72,83,88,95,100–102^

Recently, we extended the full four-component relativistic Dirac-Kohn–Sham (DKS) method, as implemented in the BERTHA code, to include environmental and confinement effects via the FDE scheme (DKS-in-DFT uFDE), see ref 73. The implementation takes advantage of the DKS formulation in BERTHA, which uses density fitting algorithms at the core of the computation (i.e., in the evaluation of the embedding potential and its matrix representation in relativistic G-spinor functions); some details will be given below.

As already mentioned, the FDE scheme has been extended to response theory since many years to access various interesting properties,^47,48,80,103,104^ such as electronic absorption^92^ and NMR shielding,^102,105^ for which FDE has been shown to perform well, as these properties are often relatively local. In a response formulation, the embedding potential and its derivatives enter the equations. If more than one subsystem is allowed to respond to external perturbations,^47,48,103,104^ the derivatives of the embedding potential introduce coupling in the subsystems’ response, just as the embedding potential couples the subsystems’ electronic structures in the ground state.

The theoretical background of subsystem TDDFT has also been reviewed in ref 81, which discusses recent advances in both theory and applications. An important development concerns the real-time versions of subsystem TDDFT, introduced by Krishtal et al.^46^ and implemented using plane waves and periodic boundary conditions. An implementation using localized basis functions was presented by De Santis et al.^56^ and more recently in ref 61. In its simplified “uncoupled” form, one considers only the response to the external applied field of a subsystem of interest (and thus the embedding potential and its derivative with respect to this subsystem’s density). While neglecting the environment response may seem a drastic approximation, good performance relative to supermolecular reference data has been obtained for the excitation energies of a chromophore in a solvent or crystal environment, even when only retaining the embedding potential.^106,107^ We will employ this framework (uFDE) in this contribution.

### 2.2 The Real-Time TDDKS Method and Its Extension to FDE Based on Density Fitting

For the theoretical foundation of the Dirac-Kohn–Sham (DKS) methodology, we refer the reader to previous works^99,108–115^ and the references therein. Here, we summarize only the main aspects of the implementation of an all-electron DKS method based on G-spinor basis sets and density-fitting techniques, as implemented in BERTHA^68^ and its Python API, PyBERTHA,^64,65^ which are important for understanding the extension of our real-time TDDKS formulation to the uncoupled FDE scheme (rt-TDDKS-uFDE) in this context.

In atomic units, and considering only the longitudinal electrostatic potential, the DKS equation is given by

8 $$\left\{c\alpha \cdot p+\beta c^{2}+v^{\left(l\right)}\left(r\right)\right\}\Psi_{i}\left(r\right)=\varepsilon_{i}\Psi_{i}\left(r\right)$$

where *c* is the speed of light in vacuum, **p** is the electron momentum, and

$$\alpha =(\begin{array}{ll} 0 & \sigma \\ \sigma & 0 \end{array})\;\mathrm{and}\;\beta =\left(\begin{array}{ll} I & 0\\ 0 & -I \end{array}\right)$$ 9

where **σ** = (σ_*x*_, σ_*y*_, σ_*z*_), σ_*q*_ is a 2 × 2 Pauli spin matrix and ***I*** is the 2 × 2 identity matrix. The longitudinal interaction term is represented by a diagonal operator borrowed from nonrelativistic theory and made up of: a nuclear potential term *v*_N_(***r***), a Coulomb interaction term *v*_H_^(*l*)^[ρ­(***r***)], and the exchange-correlation term *v*_XC_^(*l*)^[ρ­(***r***)]. We note that the Breit interaction contributes to the transverse part of the Hartree interaction and is not considered here, as we restrict ourselves to nonhybrid, nonrelativistic functionals of the electron density.

In BERTHA, the spinor solution (**Ψ**_*i*_(**r**) in eq 8) is expressed as a linear combination of G-spinor basis functions,^116^ *M*_μ_^*T*^(***r***) (*T* = *L*, *S* with *L* and *S* referring to the so-called “large” and “small” components, respectively). The G-spinors do not suffer from the variational problems of kinetic balance (see ref 117. and references therein) and, for the evaluation of multicenter integrals, retain the advantages that have made Gaussian-type functions the most widely used expansion set in nonrelativistic quantum chemistry.

Recently, the DKS method has been extended to real time, solving the real-time time-dependent DKS equation (rt-TDDKS) to investigate both linear and nonlinear properties in molecular systems. The method has been detailed by Repisky and co-workers^62,118,119^ and also by some of us;^64–66^ here, we summarize only the main points specific to our implementation in the BERTHA code. The time-dependent equation for rt-TDDKS is conveniently expressed in terms of the Liouville–von Neumann (LvN) equation, which in an orthonormal basis reads

10 $$i\frac{\partial D\left(t\right)}{\partial t}=H\left(t\right)D\left(t\right)-D\left(t\right)H\left(t\right)$$

where ***D*** (*t*) and ***H*** (*t*) are the one-electron density matrix and time-dependent DKS matrix, respectively. The time dependence of ***H*** (*t*) arises from the explicitly time-dependent external electric field and, implicitly, from the time dependence of the density matrix ***D*** (*t*) in the Coulomb and exchange-correlation terms. In BERTHA, the DKS matrix is built in atomic (AT) G-spinor basis set^116^ and defined at each time *t* as

11 $$H_{\mathrm{DKS}}^{\mathrm{AT}}=\left[\begin{array}{ll} V^{\left(\mathrm{LL}\right)}+mc^{2}S^{\left(\mathrm{LL}\right)} & c\Pi^{\left(\mathrm{LS}\right)}\\ c\Pi^{\left(\mathrm{SL}\right)} & V^{\left(\mathrm{SS}\right)}-mc^{2}S^{\left(\mathrm{SS}\right)} \end{array}\right]$$

The ***H***_*DKS*_^*AT*^ matrix is defined in terms of ***V*** ^(*TT*)^, ***S*** ^(*TT*)^, and **Π** ^(*TT*′)^ matrices being, respectively, the G-spinor basis set representation of the local potential, the overlap matrix, and the matrix of the kinetic operator (the label *TT* = *LL*, *SS* and *TT*′ = *LS*, *SL*, with *L* and *S*). The local potential ***V*** ^(*TT*)^ is given by the sum of four terms:

12 $$V^{\left(\mathrm{TT}\right)}=v^{\left(\mathrm{TT}\right)}+J^{\left(\mathrm{TT}\right)}+V_{\mathrm{xc}}^{\left(\mathrm{TT}\right)}+v_{\mathrm{ext}}\left(t\right)$$

the nuclear (***v*** ^(*TT*)^), Coulomb (***J*** ^(*TT*)^[ρ­(*t*)]), and exchange-correlation potential (***V***_*xc*_^(*TT*)^[ρ­(*t*)]) and external time dependent potential (***v***_*ext*_(*t*)).

The DKS method enables the variational incorporation of both scalar and spin–orbit interactions. However, several approximations are implicitly introduced when the Hamiltonian is used in the form defined by eq 11. The most significant approximation in the theory arises from the use of exchange-correlation functionals that depend only on the density, rather than on the relativistic four-current; this is reflected in ***V***_*xc*_^*TT*^[ρ]. Research on current-dependent functionals in a relativistic context is still at an early stage. In practice, nonrelativistic density functionals are used, even though they were not explicitly designed for relativistic calculations. Specifically, in our DKS implementation, we use the so-called “density-only” approximation, in which the exchange-correlation term depends only on the charge density (and its gradients), and not on other variables, such as spin density or magnetization,^120^ which may also be used to reparametrise the exchange-correlation potential. It is known that such a restriction limits the achievable accuracy in describing electronic transitions involving, for example, spin-flip mechanisms. Another approximation, also implicit in the exchange-correlation potential of the form ***V***_*xc*_^*TT*^[ρ­(*t*,*r*) ], is the so-called “adiabatic approximation”, which assumes that the exchange-correlation potential depends only on the density at time *t* and, by definition, cannot include any memory effects.

As already mentioned, the DKS Hamiltonian (***H***_*DKS*_^*AT*^) is evaluated in atomic basis set, whereas the LvN eq (eq 10) is represented in the orthonormal ground-state molecular spinors. The two representations are related by the simple transformation ***H*** (*t*)_*DKS*_ = ***C*** ^†^ ***H*** (*t*)_*DKS*_^*AT*^ ***C***, where ***C*** is the matrix of occupied ground-state molecular spinors (see ref 64).

The time evolution of the density matrix is given by

13 $$D\left(t\right)=U\left(t,t_{0}\right)D\left(t_{0}\right)U{\left(t,t_{0}\right)}^{†}$$

where ***U*** (*t*, *t*_0_) denotes the time-evolution operator. The use of eq 13 requires selecting a density matrix that represents the initial condition (at *t*_0_) of the molecular system. In most applications, it is customary to set the initial density as that corresponding to the ground state of the system (for details, see refs 64,73 ).

Within a finite time interval, solving the Liouville-von Neumann equation requires calculating the DKS matrix at discrete time steps and propagating the density matrix over time. In the current implementation, we use the Magnus propagator, performing matrix exponentiation exactly through matrix diagonalization. Typically, the Magnus expansion is truncated at the first order, and the time integral is evaluated using numerical quadrature, using the midpoint rule. Provided that the time interval Δ*t* is sufficiently short, the time evolution operator can be approximated as

$$U\left(t+\Delta t,t\right)\approx \exp\left[-iH\left(t+\frac{\Delta t}{2}\right)\Delta t\right]$$ 14

This approach exhibits an error proportional to (Δ*t*)^3^. The expression in eq 14 coincides with the so-called modified-midpoint unitary transform time-propagation scheme originally introduced by Li et al.^121^ The ***H*** matrix at time *t* + Δ*t*/2, where no density is available, is obtained using an iterative series of extrapolations and interpolations at each time. If this predictor/corrector procedure is converged in a self-consistent manner, the second-order midpoint Magnus propagator preserves time-reversal symmetry, which is an exact property of the equation of motion in the absence of a magnetic field. The predictor/corrector scheme is a key ingredient in preserving the numerical stability of the propagation with a range of algorithms that can be applied in this context.^122^ We use a particularly stable predictor/corrector scheme, originally proposed by Repisky et al.,^62^ and implemented in the PyBERTHA framework.^57,64,65^

From the time evolution of the system, one can obtain both linear and nonlinear properties, depending on the specific shape and strength of the external field. As an example, the dipole strength function S­(ω) is related to the imaginary part of the frequency dependent linear polarizability by

15 S(ω)=2ω3πIm⁡Tr[α(ω)]

In the linear-response regime, the components of the frequency dependent tensor are related with the Cartesian components of the electric induced dipole moment (μ̅_*p*_)­

$${\mathrm{\mu ̅}}_{p}\left(\omega \right)=\sum_{q}\alpha_{\mathrm{pq}}\left(\omega \right)E_{q}\left(\omega \right)$$

In our implementation,^64^ the external field can be chosen as an impulsive perturbation given by **E**(*t*) = *kδ*(*t*)**n**, where **n** is a unit vector representing the orientation of the field and for the δ-function we adopt its analytic representation as proposed in ref 62. Our implementation supports various field envelope shapes (e.g., Gaussian envelope); however, using an impulsive perturbation in time has the advantage of containing all frequencies (*E*(ω) = *k*), which are therefore probed simultaneously. In the case where one chooses to perturb the system along a selected Cartesian axis, *p*, the diagonal components of the polarizability tensor are proportional to the components of the induced dipole moment,

$$\alpha_{\mathrm{pp}}\left(\omega \right)=\frac{{\mathrm{\mu ̅}}_{p}\left(\omega \right)}{k}$$

that can be easily extracted from our real-time simulation via Fourier Transform of the time-dependent electric dipole moment μ⃗(*t*). Each Cartesian component p (with *p* = *x*, *y*, *z*) is given by

16 $${\mathrm{\mu ̅}}_{p}\left(\omega \right)=\frac{1}{2\pi }\int_{-\infty }^{\infty }\left(\mu_{p}\left(t\right)-\mu_{p}\left(0\right)\right)e^{i\omega t}\mathrm{dt}$$

To simulate the absorption spectra of a molecule in a sample with randomly distributed orientations, three independent simulations must be carried out, each with the perturbation applied along one of the three Cartesian axes. To extract nonlinear effects, one can apply a strong field with a specific shape.^18^ For evaluating high harmonic generation spectra using *PyBERTHA-RT*, see, for instance, ref 64.

The rt-TDDKS scheme summarized above is extended here to the subsystem density functional theory framework, specifically to FDE in the uncoupled scheme (rt-TDDKS-uFDE). We therefore consider the active subsystem, which may be subjected to a time-dependent external field, at the DKS level, while the electron density of the environment is kept frozen at its ground state value. Thus, a LvN-type equation can be solved solely for the active system, coupled to the frozen environment considered at the DFT level. Note that, although the electron density of the environment is kept frozen, the embedding potential is nevertheless time-dependent due to the time dependence of the electron density of the active system itself (ρ_I_ in eq 7 is now time-dependent). The only modification to eq 10 is in the definition of the effective Hamiltonian matrix representation, which now refers to the active subsystem (***H***_*DKS*_^*AT*,I^(*t*)). The matrix representation of the embedding potential (***V*** ^emb(TT)^(*t*)), which accounts for the effect of the environment, is added to the local potential ***V*** ^(*TT*)^ introduced in eq 12. With this substitution, the time propagation scheme remains unchanged.

As mentioned above, some of us have already presented the integration of the uFDE scheme into the DKS method for the ground state, using an efficient implementation based on density fitting and prototyping techniques, resulting in the PyBERTHA-Embed code.^73^ Here, we use a similar strategy.

The current version of BERTHA takes advantage of both density fitting algorithms^69,123,124^ and advanced parallelization techniques^66,67,71^ for the efficient evaluation of Coulomb and exchange correlation contributions to the DKS matrix. In the G-spinor basis set, the “active” system electron density is defined as

17 $$\rho_{I}\left(r\right)=\sum_{T}\sum_{\mu ,\nu }D_{\mu \nu }^{\left(\mathrm{TT}\right)}\rho_{\mu \nu }^{\left(\mathrm{TT}\right)}\left(r\right)$$

where *D*_*μν*_^(*TT*)^ represents the density in the G-spinor basis and ρ_*μν*_^(*TT*)^(***r***) are the G-spinor overlap densities. The label *TT* = *LL*, *SS* indicates that *L* and *S* refer to the “Large” and “Small” components of the G-spinor basis, respectively.

This quantity (which is a real scalar function) can be accurately approximated and linearized using a set of N_aux_ auxiliary functions.

18 $${\mathrm{\rho ̃}}_{I}=\sum_{t=1}^{N_{\mathrm{aux}}}d_{t}f_{t}\left(r\right)$$

The expansion coefficients *d*_*t*_ are worked out as the solution of the linear system, given by

19 Ad⁡=v

where ***A*** is a real and symmetric matrix, representing the Coulomb interaction in the auxiliary basis, *A*_*st*_ = ⟨*f*_*s*_ ∥ *f*_*t*_⟩ while the elements (*v*_*s*_) of the vector ***v*** are the projection of the electrostatic potential on the fitting functions, ⟨*f*_*s*_ ∥ ρ_*I*_⟩.

For the exchange-correlation ***K*** matrix, we adopt a similar strategy by solving for ***z*** in the linear system

20 Az=w

where vector ***w*** is the projection of the exchange-correlation potential (*ṽ*_xc_^(l)^[ρ̃(***r***)]) on the fitting functions

$$w_{s}=\left\langle {ṽ}_{\mathrm{xc}}^{\left(l\right)}\right|f_{s}\rangle =\int {ṽ}_{\mathrm{xc}}^{\left(l\right)}\left[\overset{\sim }{\rho_{I}\;}\left(r\right)\right]f_{s}\left(r\right)dr$$ 21

The elements of vector **w**, which involve integrals of the exchange-correlation potential, are computed numerically by the integration scheme already implemented in the code.^112^ Once the vectors ***d*** and ***z*** have been worked out, the Coulomb and the exchange-correlation contributions to the DKS matrix can be evaluated in terms of 3-center two-electron integrals *I*_*s*,*μν*_^(*TT*)^ = ⟨*f*_*s*_ ∥ ρ_*μν*_^(*TT*)^⟩

22 $${\mathrm{J̃}}_{\mu \nu }^{\left(\mathrm{TT}\right)}+{\mathrm{K̃}}_{\mu \nu }^{\left(\mathrm{TT}\right)}=\sum_{t=1}^{N_{\mathrm{aux}}}I_{t,\mu \nu }^{\left(\mathrm{TT}\right)}\left(d_{t}+z_{t}\right)$$

The extension of this scheme to include the contribution of the FDE potential, *v*_emb_^I^[ρ_I_, ρ_II_]­(***r***), is relatively straightforward. The evaluation of the embedding potential matrix (*Ṽ*_*μν*_^*emb*(*TT*)^) can strictly follow a procedure similar to that employed for the exchange-correlation^125^ term, and it is given by

$${Ṽ}_{\mu \nu }^{\mathrm{emb}\left(\mathrm{TT}\right)}=\frac{\partial E_{\mathrm{int}}\left[{\mathrm{\rho ̃}}_{I},\rho_{\mathrm{II}}\right]}{\partial D_{\mu \nu }^{\left(\mathrm{TT}\right)}}=\int \frac{\delta E_{\mathrm{int}}\left[{\mathrm{\rho ̃}}_{I},\rho_{\mathrm{II}}\right]}{\delta {\mathrm{\rho ̃}}_{I}\left(r\right)}\frac{\partial {\mathrm{\rho ̃}}_{I}\left(r\right)}{\partial D_{\mu \nu }^{\left(\mathrm{TT}\right)}}dr$$ 23

The matrix elements, *Ṽ*_*μν*_^*emb*(*TT*)^, is expressed as a linear combination of the three-index coulomb repulsion integrals as^56^

24 $${Ṽ}_{\mu \nu }^{\mathrm{emb}\left(\mathrm{TT}\right)}=\sum_{t=1}^{N_{\mathrm{aux}}}I_{t,\mu \nu }^{\left(\mathrm{TT}\right)}c_{t}$$

The expansion coefficients (*c*_*t*_) are the elements of vector ***c***, solution of the linear system ***Ac*** = ***g***, whre vector ***g*** is the projection of the embedding potential, *v*_emb_^I^[ρ̃_I_, ρ_II_]­(***r***), on the fitting functions.

$$g_{s}\left(t\right)=\left\langle {ṽ}_{\mathrm{emb}}\right|f_{s}\rangle =\int {ṽ}_{\mathrm{emb}}\left[\overset{\sim }{\rho_{I}\;}\left(t\right),\rho_{\mathrm{II}}\left(r\right)\right]f_{s}\left(r\right)dr$$ 25

The embedding potential contribution is efficiently evaluated in a single step together with the Coulomb and the exchange-correlation ones, and finally added to the DKS matrix, *J̃*_*μν*_^(*TT*)^ + *K̃*_*μν*_^(*TT*)^ + *Ṽ*_*μν*_^*emb*(*TT*)^ = ∑_*t* = 1_^*N*_aux_^ *I*_*t*,*μν*_^(*TT*)^(*d*_*t*_ + *z*_*t*_ + *c*_*t*_). As in the electronic ground state case, where changes in the active subsystem density require updating ***V*** ^emb^ during the SCF cycles, the time propagation of the electron density introduces a time dependence in ***V*** ^emb^ even though the environment densities remain frozen due to the use of the uncoupled scheme. This means that the embedding potential matrix, ***V*** ^emb^, must be updated at each time step. We will show that the numerical noise associated with constructing the embedding potential in this scheme does not affect the numerical stability of the propagation.

### 2.3 Fast Prototyping and Implementation

Here, we outline the computational strategy we have adopted to implement the rt-TDDKS-uFDE scheme. The developed Python program PyBERTHA-Embed-RT and the related modules (PyBERTHA, PyBERTHA-Embed and PyBERTHA-RT) are freely available under GPLv3 license at ref 65. A data set collection of computational data including geometries of the systems, numerical data, parameters and job input instructions used to obtain the results of Section 3, is available and can be freely accessed at the Zenodo repository, see ref 126.

In our workflow, we have combined the real-time TDDKS reference procedure, thus the PyBERTHA-RT module,^64,65^ already implemented within the BERTHA code thanks to its Python API PyBERTHA,^64,65,67^ with the FDE computational core that we developed in the PyBERTHA-Embed module.^65,73^ Both PyBERTHA-RT and PyBERTHA-Embed modules are described in detail in ref 64. and ref 73., respectively.

The PyBERTHA-RT code is based on PyBERTHA, a Python^127^ API that has contributed to improving both the usability and interoperability of the BERTHA code.^64,66,67,73^ All the basic kernel functions written in FORTRAN are collected in a single Shared Object (SO) capable of performing both serial and parallel calculations using both OpenMP-based operations^66^ or hybrid OpenMP/OpenACC ones (i.e., CPU and GPU).^71^ A FORTRAN module, named *bertha*_*wrapper*, contains a class implementing all the methods needed to access the basic quantities, such as energy, density, DKS and overlap matrices, as well as other functions that can be easily implemented. Finally, the main PyBERTHA^65^ module has been developed using the *ctypes* Python module. This module provides C-compatible data types and allows calling functions collected in shared libraries. Thanks to this Python API, the Magnus propagator scheme in PyBERTHA-RT is written entirely in Python, using the *numpy* or *cupy* modules for the required linear algebra operations^64,71^ (matrix–matrix multiplication and diagonalization).

The PyBERTHA-Embed module is based on PyBERTHA and on general PyEMBMOD module. A specific class inside the PyEMBMOD module allows to well isolate all the FDE data and operations increasing the level of abstraction. The module is used to manage all the required quantities for the generation of the embedding potential, that is *v*_emb_[ρ̃_*I*_, ρ_*II*_(***r***)]. It has been engineered in a such manner that all details of the FDE low-lying implementation are completely transparent from the PyBERTHA side. This has the advantage that all future developments and/or integration of the FDE scheme (g.e. using DKS theory also for the environment DKS-in-DKS FDE) will not affect the PyBERTHA-Embed code, i.e., it will remain completely unchanged. In particular in this first version, the PyEMBMOD module can handle the basic procedures which are based on the use of PyADF,^74,75,128^ PyEmbed module,^75,129,130^ and the XCFun library^77^ to evaluate nonadditive exchange-correlation and kinetic energy contributions on user-defined integration grids. This approach gave us both the advantages of the code reusability and clearly made the debugging phase in the development of software straightforward. Finally, we mention that PyADF is not specific to a single program, but works with a number of different quantum chemistry codes, in this context we use it with ADF program.^90^

An example of the interoperability achieved between different modules is presented in Figure 1, where a basic workflow of our rt-TDDKS-uFDE implementation in PyBERTHA-Embed-RT is shown. The code accepts several parameters that define the molecular fragments and specify the details of the calculations: geometry, G-spinor basis set, and exchange-correlation functional used in the DKS calculation for the active system. Similarly, it requires the geometry, basis set, exchange-correlation functional, and other details for the environmental system, which, in this context, is handled using the ADF code via PyADF. It also takes the information required for real-time propagation. All of these options are already available in the PyBERTHA-RT code. These include, for example, the definition of the external electric field function, its direction, the total simulation time, and the threshold for the predictor/corrector scheme used in the Magnus propagator. An additional option, which can now be set in a PyBERTHA-Embed-RT run, specifies the frequency at which the embedding potential is updated during propagation. In all numerical examples reported below, we update the embedding potential at each time step of the propagation.

**Figure 1:**
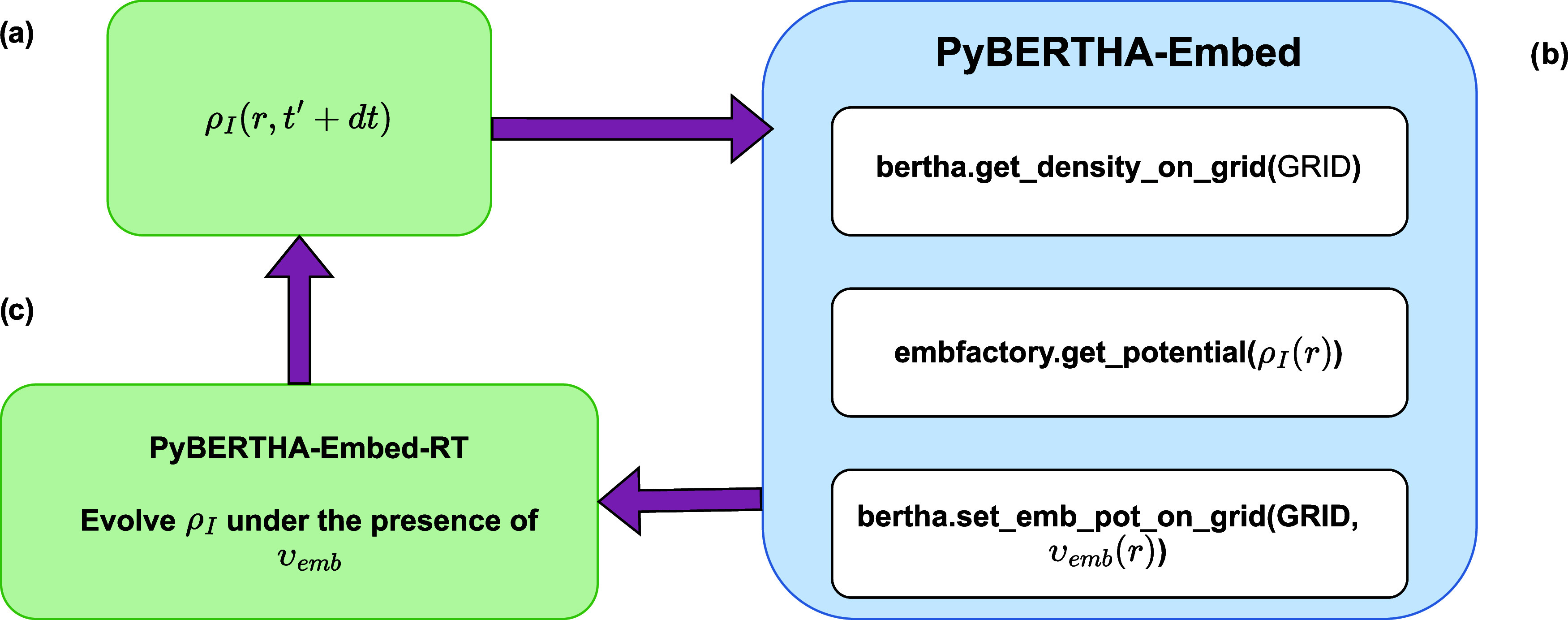
Basic real-time DKS-uFDE flow in PyBERTHA-Embed-RT: (a) numerical representation of ρ̃(*r*, *t*′ + *dt*) on the grid; (b) PyBERTHA-Embed classes are used to calculate the embedding potential; (c) time-evolution step.

The electron density of an active system is obtained via a DKS calculation using PyBERTHA while the electron density, the Coulomb potential and nonadditive terms of the environment are managed using PyADF. These quantities are mapped on a common numerical grid and used in PyEMBMOD to evaluate the embedding potential. We refer the interested reader to refs 56,73 , where some of us presented the workflow implemented in PyBERTHA-Embed.

The complete workflow can be summarized as follows: there is an initial phase in which: (i) a single self-consistent DKS calculation is performed on the active system to obtain an initial electron density (ρ̃_*I*_); (ii) the PyEMBEDMOD module initializes and manages the calculation of the frozen environment, using PyADF in combination with the ADF code. This module also defines the numerical grid that will be used to represent the key quantities in the procedure. In this phase, the SCF calculation is carried out on the environment systems, and the first two terms of the embedding potential (the nuclear potential and the Coulomb potential of fragment *II*, see eq 7), which remain unchanged during the propagation, are mapped onto the numerical grid. Once the electron density of the active system (ρ̃_*I*_(*r*)) has been calculated at the DKS (or rt-TDDKS) level, then its G-spinor representation is converted to the numerical representation on the grid (i.e., *get*_*density*_*on*_*grid* in Figure 1). Then we evaluate the corresponding nonadditive embedding potential and return the embedding potential (*v*_*emb*_(*r*_*i*_)) on the grid as a numpy array (i.e., *get*_*potential* in Figure 1). Afterward, starting from the numerical representation *v*_*emb*_(*r*_*i*_) and the numerical grid, we build its G-spinor matrix representation (*Ṽ*_*μν*_^*emb*(*TT*)^) by projecting the embedding potential onto the density fitting basis set via numerical integration (see eq 24 and eq 25) (see *set*_*embpot*_*on*_*grid* in Figure 1).

The *Ṽ*_*μν*_^*emb*(*TT*)^ matrix is then added to the full ***H***_*DKS*_^*AT*^ and used to converge the ground state DKS calculation and subsequently during the time propagation of the rt-TDDKS-uFDE. Note that in this latter case, the procedure described above (see also Figure 1, panel b) is performed at each time step of the propagation because the electron density of the active fragment (ρ_*I*_(*t*)) is time dependent, which is reflected in the time dependence of the embedding potential (*v*_emb_^I^[ρ_I_(*t*), ρ_II_]­(***r***)).

## 3 Absorption Spectra of PbX_2_-GBL systems (X = Cl, I)

Here, we use the newly developed *PyBERTHA-Embed-RT* code to simulate the absorption spectra of PbCl_2_ and PbI_2_ molecules interacting with an increasing number of solvent molecules, up to 32, specifically γ-butyrolactone (GBL). The solution chemistry of PbCl_2_ and PbI_2_ is relevant to the synthesis of lead-halide perovskites, which are widely investigated for applications in solar cells and other electronic devices.^76^ Solution processing typically involves mixing a lead-halide precursor with a halide salt in a strongly coordinating solvent (e.g., GBL or others), followed by the formation of the perovskite solid phase upon solvent elimination. Chlorine-containing precursors are known to increase perovskite grain size and crystallinity, leading to more homogeneous perovskite thin films and improved charge-carrier transport. The different behaviors of PbCl_2_ and PbI_2_ in solution therefore contribute to controlled perovskite synthesis. A detailed account of these precursors in solution was provided by Kaiser et al.,^76^ combining ab initio molecular dynamics, experimental UV–vis absorption measurements, and frequency-domain TDDFT simulations employing the B3LYP functional. One key finding from this prior study is that PbCl_2_ is generally less solvated than PbI_2_, consistent with a higher lead-halide bonding energy in PbCl_2_: chlorine acts as a stronger ligand and limits coordination by solvent molecules.

Before presenting our results, we emphasize that the primary goal of these simulations is to demonstrate the numerical stability of the new implementation and its potential applicability to complex chemical systems. The present calculations are not intended to enable a strict comparison with experimental UV–vis measurements. Two main factors limit quantitative agreement: (i) the use of GGA exchange-correlation functional (due to current implementation constraints), which is expected to underestimate the transition energies; (ii) the use of a single AIMD snapshots, which neglects nuclear dynamics and thermal averaging. The error associated with approximation (i) may be reduced by using hybrid or range-separated hybrid functionals, which are known to yield more accurate transition energies, while the limitation in approximation (ii) can be alleviated by averaging the absorption spectra over multiple nuclear configurations. Furthermore, as we use FDE in the uncoupled scheme, we neglect the polarization response of the environment upon excitation/perturbation, cannot accurately describe electronically excited states with significant contributions from both the active subsystem and the environment. Within uFDE, the smaller the active subsystem, the less one can expect to reproduce all features of a supermolecular calculation, as only a limited portion of the occupied and virtual spaces is represented.

The geometries used in this study were extracted from a snapshot of the molecular dynamics simulation reported in the literature by some of the authors^76^ and are provided explicitly in the referenced data set.^126^ As an example, Figure 2 shows the structures for PbCl_2_ interacting with 1, 2, 3, and 32 molecules of GBL. For the active systems (PbCl_2_ and PbI_2_) we used DKS level of theory and Dyall’s basis sets, specifically *dyall.v2z* and *dyall.v3z*,^131^ which are designed for all-electron relativistic calculations for the whole periodic table. Alongside these orbital basis sets, we used the auxiliary basis set Gen-n2-v1 and evaluated the effect of two more accurate auxiliary fitting basis sets, Gen-n3-v1 and Gen-n3-v2. These auxiliary basis sets were generated through an algorithm we have recently developed in ref 124. For all real-time simulations, we used 20 000 time steps with a time step of 0.15 au for a total time of 3000 au (72 fs). An analytic δ­(*t*) was used as an external perturbation with a field intensity of 5 × 10^–5^ a.u. In all cases, the PBE exchange-correlation functional was applied within the density-only approximation (see above for more details). The embedding potential, originated by the GBL solvent molecules, has been updated at each time step of the real-time evolution and has been obtained using PyADF (and the PyBerthaEmbed module) in combination with ADF packages^90^ and the TZ2P basis set. The absorption spectra, related to the dipole strength function *S*(ω) (eq 15), have been obtained using the Fourier Transform of time-dependent induced dipole moment of the active system molecule using Pad’ approximation,^132^ in combination with a Gaussian damping function with an exponent of 1 × 10^–6^.

**Figure 2:**
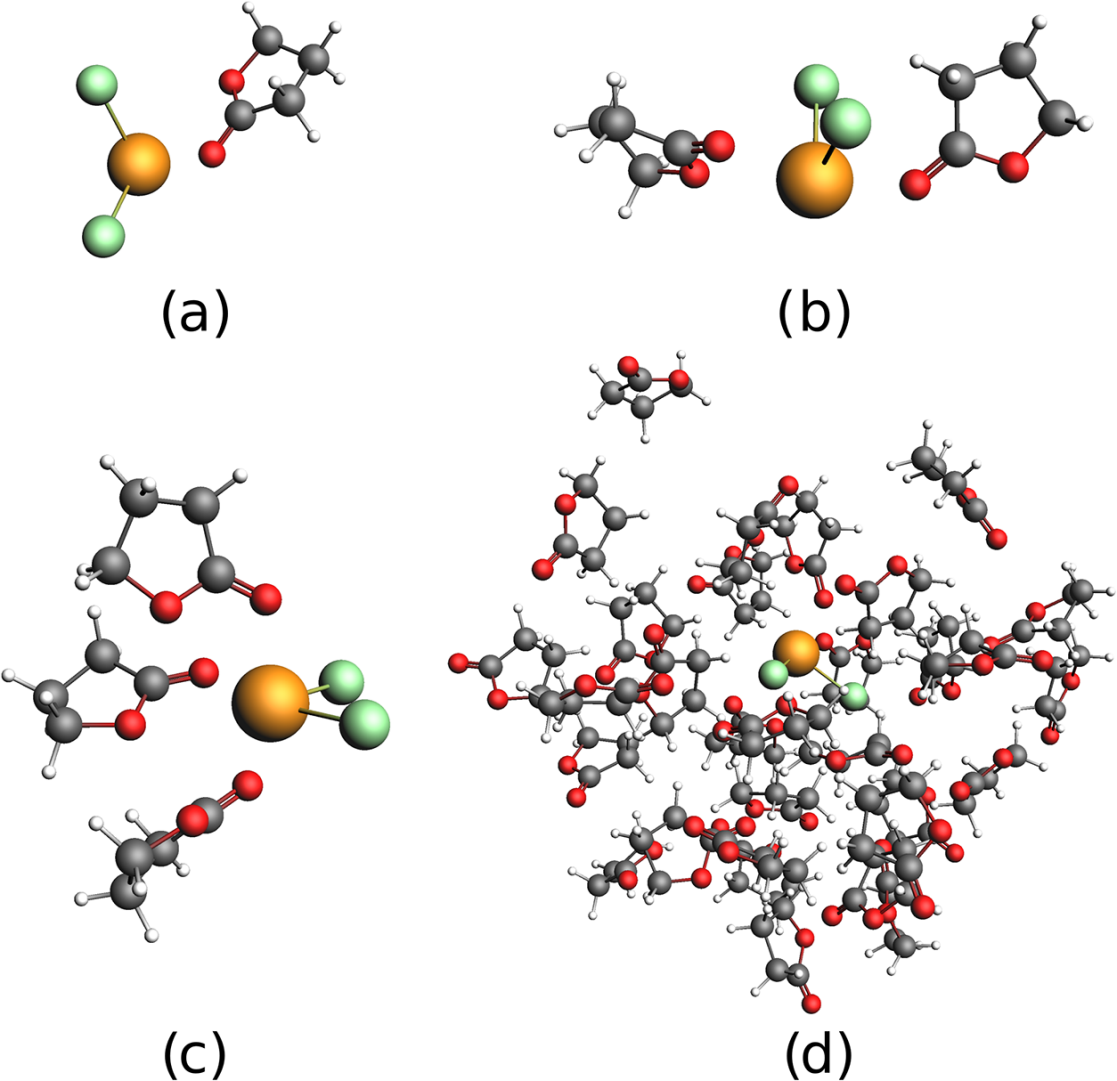
Geometric structures used in this work, based on a snapshot of molecular dynamics simulations by Kaiser et al.:^76^ (a) PbCl_2_-GBL system; (b) PbCl_2_-(GBL)_2_ system; (c) PbCl_2_-(GBL)_3_ system; and (d) PbCl_2_-(GBL)_32_ system. For both PbI_2_ and PbCl_2_ (not shown), the systems (a)–(c) were obtained from the full system (d) by selecting the nearest GBL neighbors to the respective lead halide.

As previously mentioned, density fitting is central to our implementation. It is used in the evaluation of the Dirac-Kohn–Sham matrix during the initial SCF procedure, throughout the real-time evolution, and in the evaluation of the G-spinor matrix representation of the FDE potential. Therefore, we first assess the impact of using different density fitting auxiliary basis sets of increasing accuracy. Data are reported for PbCl_2_ (active system) interacting with a single molecule of GBL (environment) in Figure 3 (upper panel). For a consistent comparison, we have also included the spectrum obtained for the free PbCl_2_ molecule at the same geometry (see Figure 3, lower panel). The inclusion of a single molecule of GBL via uFDE scheme reduces the symmetry and increases the number of electronic states, resulting in a much richer spectrum than that of the isolated PbCl_2_. In the low-energy region, the solvent induces a pronounced blue shift of the first transition (approximately 0.5 eV) for the specific configuration considered here. It is noteworthy that the effect of the auxiliary fitting basis sets is almost negligible for the isolated PbCl_2_. For this system, the three auxiliary fitting basis sets produce nearly indistinguishable spectra, and the smallest auxiliary basis set used (Gen-n2-v1) is as accurate as the larger one, Gen-n3-v2. However, for the embedded system, the smallest auxiliary basis set tends to shift the spectrum slightly to higher energy (by about 0.05 eV) compared to the reference data (obtained using Gen-n3-v2). This small discrepancy in the spectrum suggests that, to maintain high accuracy, the inclusion of the embedding potential requires an auxiliary fitting basis with sufficient flexibility to accurately project the embedding potential (see eq 25).

**Figure 3:**
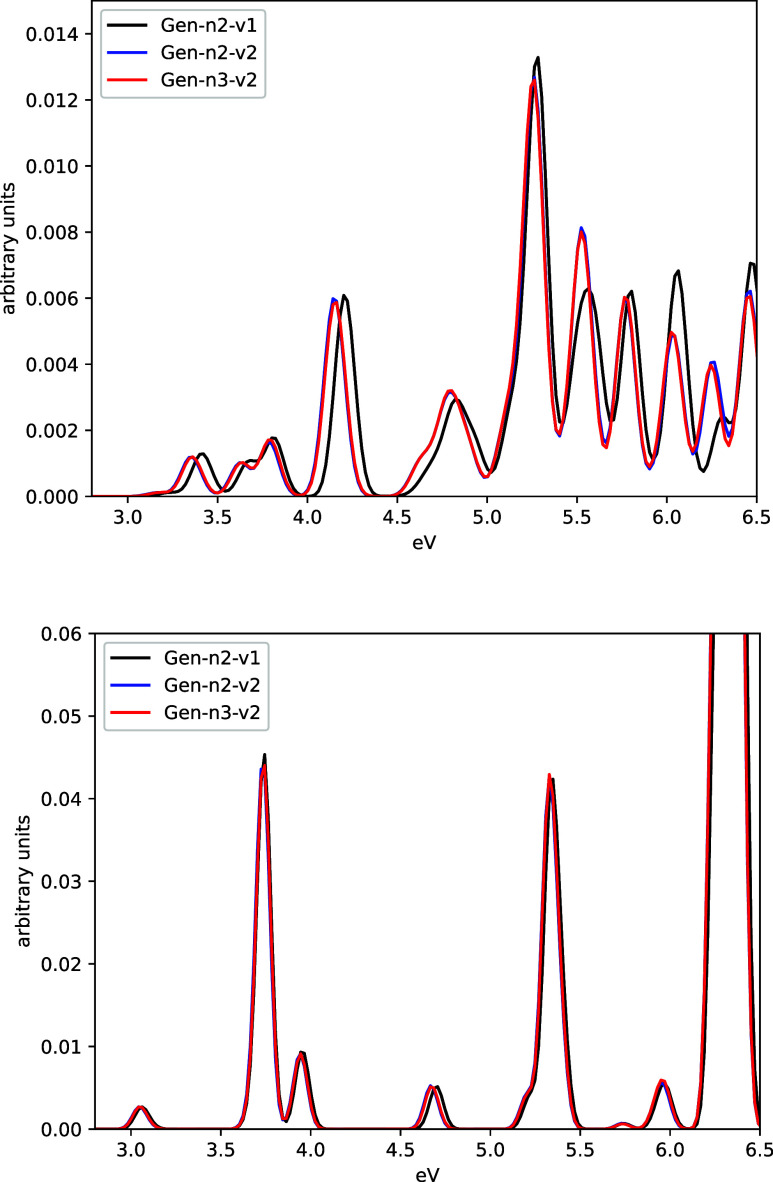
Absorption spectrum (Fourier Transform of the α_*xx*_ component) for the PbCl_2_ molecule with/without (upper/lower panel) embedded with one molecule of GBL. Data are reported using different auxiliary basis sets with increasing accuracy (see text for details).

In Figure 4, we examine the effect of the G-spinor basis sets on the resulting spectrum for the PbCl_2_ molecule in the presence of one solvent GBL molecule. The effect, passing from *dyall.v2z* to *dyall.v3z*, is relatively small at low energies (see upper panel), with differences below 0.05 eV, while at energies above 5 eV (see lower panel), the effect becomes more significant; the spectrum obtained using *dyall.v3z* basis clearly shows more electronic states due to its increased flexibility. Nevertheless, it is important to note that the numerical stability of our real-time DKS simulation, including the time-dependent embedding potential, is not compromised when using a more flexible and significantly larger principal basis set.

**Figure 4:**
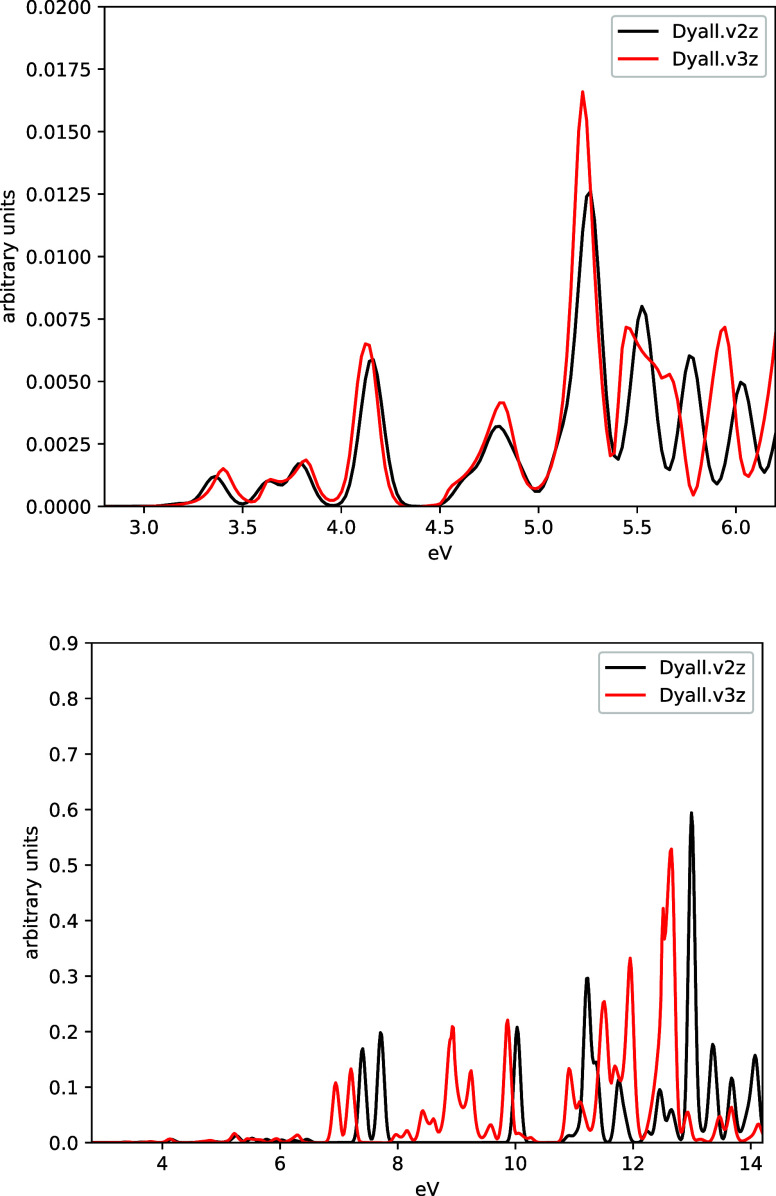
Absorption spectrum (Fourier transform of the α_*xx*_ component) for PbCl_2_ embedded with one molecule of GBL. Data are reported using different G-spinor basis sets (namely, dyall.v2z and dyall.v3z; see text for details). The energy range extends up to 6.2 eV (upper panel) and up to 14.2 eV (lower panel), so that the lower-intensity features appearing between 3 and 6.2 eV can be properly visualized.

To demonstrate the applicability of our method to more complex systems, we investigated how the absorption spectra of PbCl_2_ and PbI_2_ evolve as the number of GBL solvent molecules increases, considering *n* = 1, 2, 3, and 32 (see Figures 5 and 6). All spectra were obtained from the isotropic component of the frequency-dependent dipole polarizability (eq 15). For each system, three independent simulations were performed, applying perturbations along the three Cartesian directions, and induced dipole components were combined to obtain the isotropic response. The all-electron *dyall.v2z* basis set, in combination with the Gen-n2-v2 auxiliary fitting set,^124^ was employed. In all cases, the simulations were found numerically stable and well converged.

**Figure 5:**
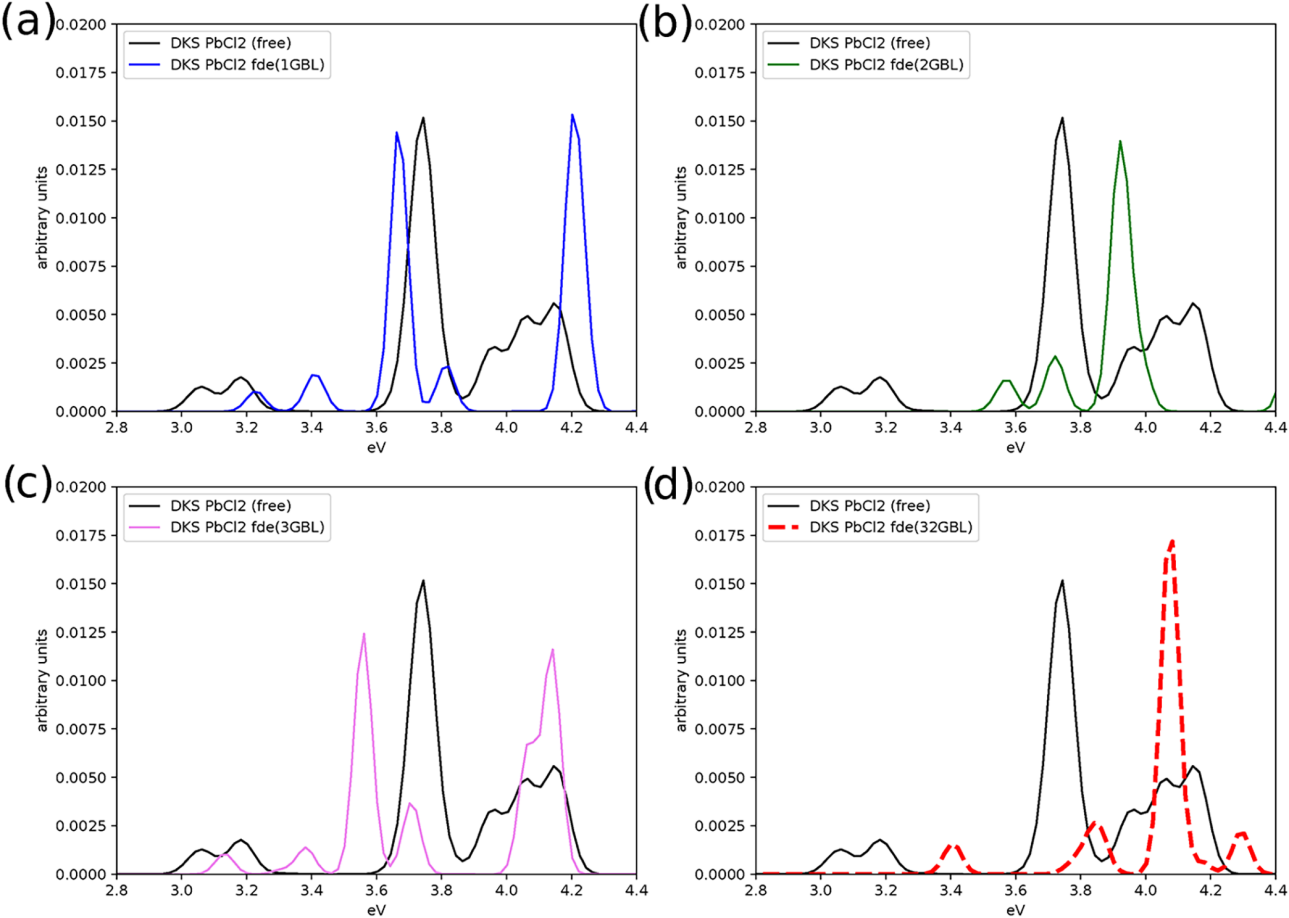
Effect on the absorption spectrum due to the interaction of PbCl_2_ with an increasing number of the GBL molecules (1, 2, 3, and 32 molecules in the panel (a)–(d), respectively). The spectrum of the isolated PbCl_2_ (denoted by “free”) has also been reported for comparison.

**Figure 6:**
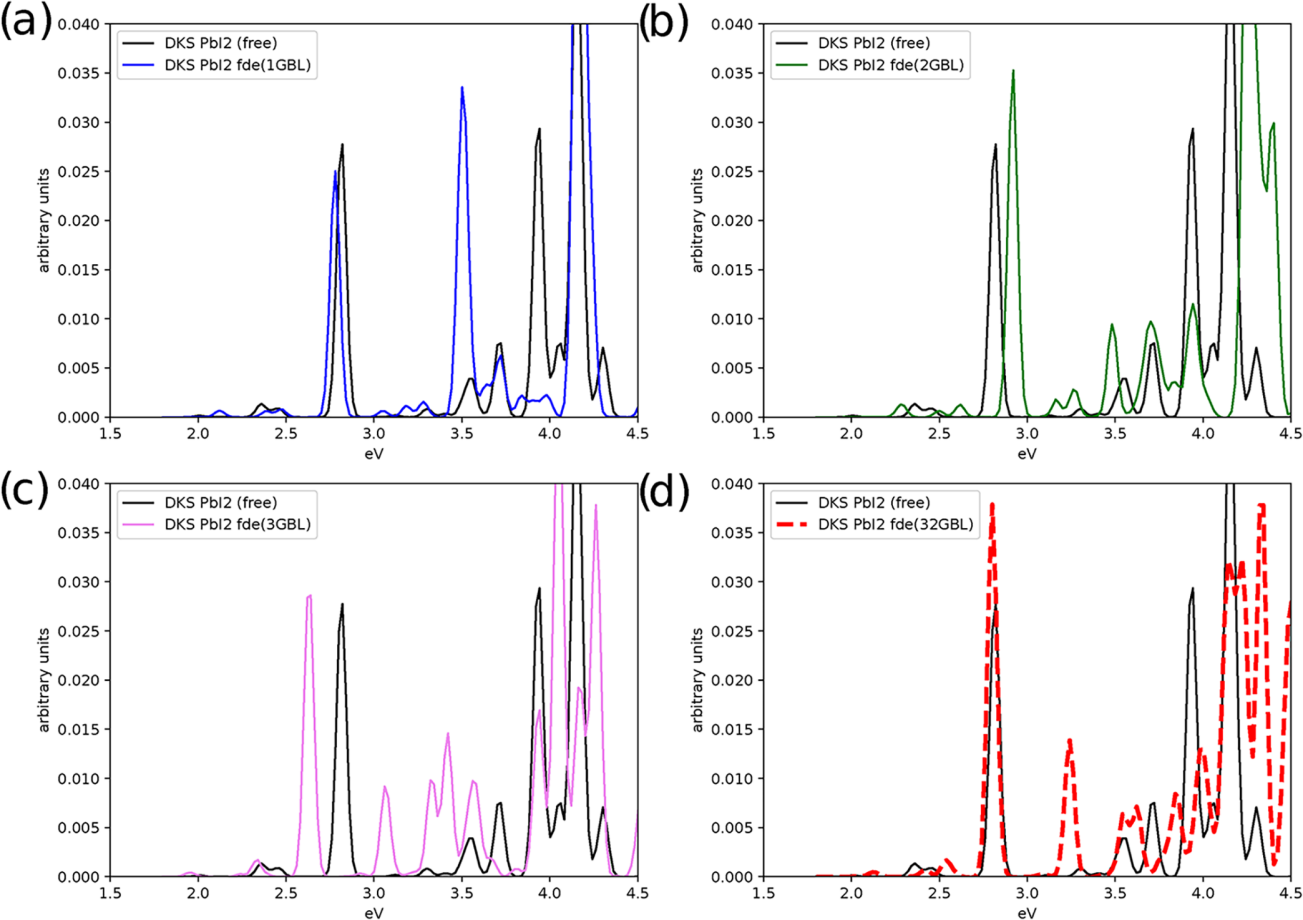
Effect on the absorption spectrum due to the interaction of PbI_2_ with an increasing number of the GBL molecules (1, 2, 3, and 32 molecules in panel (a), (b), (c), and (d), respectively). The spectrum of the isolated PbI_2_ (denoted by “free”) has also been reported for comparison.

For the PbCl_2_ series, the lowest-energy transition exhibits a blue shift relative to the isolated molecule but does not appear to vary systematically with the number of solvent molecules (0.2, 0.6, 0.15, and 0.35 eV for 1, 2, 3, and 32 GBL molecules respectively). The second feature is separated from the first one by roughly the same energy in the 1GBL and 2GBL systems, but their splitting increases when going from 2 to 3 GBL molecules, and further increases from 3 GBL to 32 GBL molecules. For the third feature, appearing near 3.8 eV in the isolated molecule and which is significantly more intense than the first two, we observe a red shift for the 1GBL and 3GBL and a blue shift for the 2GBL one, roughly in line with how the peak maxima the theoretical results shown in Figure 4 of ref 76. change with the number of GBL molecules. The blue shift of this feature is the most significant for the 32GBL case. Taken together, these changes in spectra highlight the importance of incorporating both first- and outer solvation shells, even if represented through an effective embedding potential, as done here.

In the PbI_2_ series, consistent with experimental observations,^76^ electronic transitions occur at lower energies (Figure 6) than for PbCl_2_. The first excited state of the isolated PbI_2_ molecule appears around 2.0 eV, compared with approximately 3.0 eV for PbCl_2_. Solvation induces an overall shift of approximately 0.3 eV, with PbI_2_(3GBL) exhibiting a slight red shift. Notably, the lowest-energy transition for PbI_2_(1GBL) nearly coincides with that of PbI_2_(32GBL), but there are significant differences between the isolated and solvated systems for energies above 3.0 eV, not only in terms of peak positions but also in intensities. Given that in all calculations the active subsystem has the same structure, these results again highlight the importance of incorporating molecules in the first and second solvation shells, albeit through an effective potential, as done here.

For completeness, Figure 7 compares the absorption spectrum of PbI_2_[1GBL] obtained using the uFDE (PbI_2_ active; GBL environment) with that from a supermolecular calculation. The agreement is reasonably satisfactory for the lowest-energy states and those above 3.5 eV. In contrast, the spectral region between 2.5 and 3.4 eV shows significant deviations in both peak positions and intensities. These differences are likely due to the approximations inherent in the current uncoupled FDE setup and indicate that the chosen active subsystem is too small to describe electronic states that are delocalized over both the active system and the environmental fragment. This observation suggests that a more realistic description would require the inclusion of at least the first solvent shell, with the remaining environment treated within the uFDE framework. At present, such an approach is computationally demanding and would likely benefit from the GPU-accelerated version of the code presented in ref 71.

**Figure 7:**
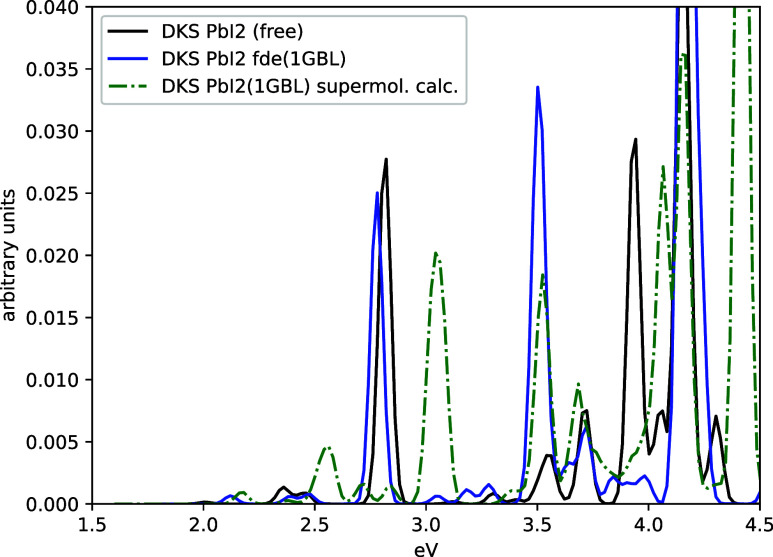
Comparison of the absorption spectrum of PbI_2_[1GBL] using the uFDE scheme, with PbI_2_ as the active system and GBL as the environment (solid blue line), and supermolecular calculation (dashed green line). The spectrum of the isolated PbI_2_ (solid black line) has also been reported for comparison.

Before concluding, it is interesting to comment on the computational cost associated with incorporating the FDE scheme in our real-time DKS implementation. The timings reported here were obtained using the dyall.v2z basis in combination with the GEN-n2-v1 auxiliary basis set (with results obtained with the GEN-n2-v3 auxiliary basis reported in parentheses), on an Intel­(R) Xeon­(R) CPU E5–2683 v4­(2.10 GHz) processor using 32 threads. Table 1 summarizes the total wall time per simulation time step. The computational cost associated with the propagation of the active subsystem time propagation (*t* ^*a*^) is largely independent of the number of environmental molecules, and increases only slightly due to the larger total number of grid points required to evaluate the active system density on the full grid (see *t* ^*b*^). In our implementation, a global grid is defined for the entire system (active subsystem plus environment), thus, the total number of grid points increases with the number of GBL molecules included in the environment. As previously mentioned, the mapping of the environment’s electron density and Coulomb potential, obtained from ADF, onto the numerical grid is performed once at the beginning of the simulation. The wall time required for this initialization step is also reported in Table 1 (*t* ^*c*^).

**Table 1:** Timing (s) for the PbCl_2_ Molecule with *n* Molecules of GBL

*n*	t^a^	t^b^	t^c^
0	5.4		
1	7.6	0.02	1.1
1	(10.1)	(0.04)	(1.1)
2	9.0	0.03	1.9
3	9.1	0.05	2.7
32	9.9	0.49	24.3

a Total average time for a single time step.

b Fitted density on the grid.

c Initialization time, this involves mapping the environment’s static properties to the grid. Data have been obtained using dyall.v2z basis in combination with the GEN-n2-v1 auxiliary basis set (in parentheses data obtained using GEN-n2-v3 auxiliary basis set.).

## 4 Conclusions and Perspectives

In this work, we presented an extension of the relativistic four-component real-time Dirac-Kohn–Sham (rt-TDDKS) method to include environmental effects via the Frozen Density Embedding (FDE) scheme. This was achieved by developing a unified framework that integrates the Python APIs of PyBERTHA and the PyADF scripting framework, resulting in an efficient and interoperable computational tool, namely PyBERTHA-Embed-RT.

Our implementation uses an uncoupled FDE approach in which only the active subsystem evolves in time, while the environment remains frozen in its ground state. We have shown that incorporating the FDE embedding potential through a density-fitting scheme does not compromise the numerical stability of the time-propagation algorithm. This was validated through systematic tests on the absorption spectrum of PbCl_2_ in the presence of the frozen density embedding potential, which produced robust and well-converged results with respect to both the primary G-spinor and auxiliary fitting basis sets.

As a practical demonstration, we investigated the absorption spectra of lead halide perovskite precursors, PbCl_2_ and PbI_2_, treated as active systems and solvated by increasing numbers of GBL molecules. The simulations successfully captured several solvent-induced effects, including the observed blue shift in the lower-energy bands as the number of solvent molecules increased. These results highlight the capability of the method to provide insights into the solution-phase chemistry of complex systems where both relativistic and environmental effects play an essential role.

By construction, the uncoupled FDE approach implemented here neglects the polarization response of the environment upon perturbation and, therefore, cannot accurately describe electronically excited states with significant contributions arising from changes in the environment. A comparison with a supermolecular calculation for PbCl_2_(1GBL) reveals features in the absorption spectrum that are not reproduced by the uFDE calculations with PbCl_2_ as the active subsystem. This suggests that, for a more accurate description, at least a solvation shell should be included in the active system of the uFDE scheme.

Looking ahead, the present framework provides a solid foundation for several promising extensions. A natural next step is the development of a coupled FDE scheme, in which the environment is allowed to respond dynamically to external fields. Such an approach would enable the study of more complex phenomena, such as intersubsystem charge-transfer excitations. Moreover, the proven stability of the real-time approach makes it particularly suitable for exploring the nonlinear optical properties of molecules in solution, an area of significant scientific interest.

The predictive power of the current implementation is also limited by the approximations used in the exchange-correlation functional, which restrict the quantitative accuracy of the computed absorption spectra relative to experiment. Future developments incorporating exact exchange as well as hybrid or long-range-corrected functionals are expected to improve predictive accuracy and fully exploit the relativistic effects, including spin–orbit coupling, offered by the DKS formalism. Furthermore, extending the present implementation to fully exploit GPU acceleration, which we have already demonstrated in the rt-TDDKS context,^71^ would be a valuable direction for improving performance and enabling larger-scale simulations. In conclusion, PyBERTHA-Embed-RT is a powerful and flexible computational framework and represents an important step toward accurate real-time simulations of molecular systems containing heavy elements embedded in complex environments.

## Data Availability

The geometries of all systems considered in this paper have been made publicly available via the Zenodo repository, see ref 126.
